# Early glycaemic variability increases 28-day mortality and prolongs intensive care unit stay in critically ill patients with pneumonia

**DOI:** 10.1080/07853890.2022.2128399

**Published:** 2022-10-07

**Authors:** Seong Ho Kim, Ji Young Kim, Eun Song Kim, Il Rae Park, Eun Yeong Ha, Seung Min Chung, Jun Sung Moon, Ji Sung Yoon, Kyu Chang Won, Hyoung Woo Lee

**Affiliations:** a College of Medicine, Yeungnam University, Daegu, Republic of Korea; b Department of Internal Medicine, Division of Endocrinology and Metabolism, Yeungnam University College of Medicine, Daegu, Republic of Korea

**Keywords:** Diabetes, glycaemic variability, coefficient of variation, intensive care unit, pneumonia

## Abstract

**Objective:**

This study aimed to evaluate the effect of early glycaemic variability (GV) on 28-day mortality in critically ill patients with pneumonia.

**Patients and methods:**

This single-centre retrospective study included patients admitted to the intensive care unit (ICU) due to pneumonia between 2018 and 2019. A total of 282 patients (mean age, 68.6 years) with blood sugar test (BST) results measured more than three times within 48 h after hospitalization and haemoglobin A1c (HbA1c) levels recorded within 2 months were enrolled. Coefficient of variation (CV) was calculated using the BST values. The effects of GV on 28-day mortality and prolonged ICU stay (>14 days) were also assessed.

**Results:**

The mean age was 60.6 years (male to female ratio, 2.5:1). The 28-day mortality rate was 31.6% (*n* = 89) and was not different according to the presence of diabetes (DM *vs.* non-DM) or HbA1c levels (≥7.5 *vs.* <7.5%; both *p* > .05). However, the mortality rate was significantly higher in patients with high GV (CV ≥ 36%) than in those with low GV (CV < 36%; 37.5 *vs.* 25.4%, *p* = .028). The risk of mortality in patients with high GV was prominent in the subgroups with DM or low HbA1c levels. Among the surviving patients (*n* = 193), 44 remained in the ICU for more than 14 days. Compared to low GV, high GV was associated with a higher rate of prolonged ICU stay, although not statistically significant (27.8 *vs*. 18.5%, *p* = .171). After adjusting for the severity of illness and treatment strategy, CV was an independent risk factor for 28-day mortality (hazard ratio [HR], 1.01, *p* = .04) and prolonged ICU stay (odds ratio, 1.02; *p* = .04).

**Conclusions:**

High GV within 48 h of ICU admission was associated with an increased 28-day mortality risk and prolonged ICU stay. Early phase GV should be carefully managed in critically ill patients with pneumonia.

**KEY MESSAGES**
- The presence of diabetes or HbA1c alone is insufficient to predict 28-day mortality and prolonged ICU stay in critically ill patients with pneumonia.
- High glycaemic variability (GV) within 48 h of ICU admission increases 28-day mortality and prolongs ICU stay, which is consistent after adjusting for severity of illness and treatment strategy.
- Patients with high GV, especially those with DM or low HbA1c levels (<7.5%) should be more carefully treated to reduce mortality.

## Introduction

Diabetes mellitus (DM) is a chronic disease characterized by high blood glucose levels owing to decreased insulin secretion or function. Diabetes has a high mortality rate due to complications caused by chronic hyperglycaemia, including microvascular and macrovascular complications [1]. Diabetes also has a negative effect on the lungs because of the abundant vascular and connective tissues (collagen and elastin) [2]. Particularly, changes in collagen and elastin occur due to the nonenzymatic glycosylation of proteins induced by chronic hyperglycaemia, resulting in pulmonary complications [3,4]. In fact, previous studies have shown that patients with DM are vulnerable to asthma, chronic obstructive pulmonary disease and pneumonia due to decreased lung function [5]. Furthermore, patients with DM are prone to infectious diseases due to reduced blood flow caused by microvascular complications [6,7], and related studies have shown that patients with DM have a poor prognosis in community-acquired pneumonia [8].

In addition, recent studies have found that high glycaemic variability (GV) is also a serious risk factor for high mortality rates in patients in intensive care units (ICUs), similar to chronic hyperglycaemia [9–11]. Furthermore, since GV in the early stages of hospitalization reflects the physiological response to stress, it has been suggested as a parameter for predicting prognosis in acute patients [12,13]. However, studies on GV have not yet been actively conducted, particularly studies on the association between GV and mortality of pneumonia, a single disease.

Therefore, this study aimed to investigate the relationship between early GV during the first 48 h and mortality in patients admitted to the ICU due to pneumonia. Moreover, the effects of GV on prolonged ICU stay were analysed.

## Patients and methods

### Study participants

This retrospective cohort study included 404 patients admitted to the ICU of Yeungnam University Hospital for pneumonia between January 2018 and December 2019. Individuals with pneumonia were selected according to the Korean Standard Disease Code (KCD-7-based International Classification of Diseases, 10th revision (ICD-10); ICD code: J09–J18). Among them, 119 patients whose blood sugar test (BST) values were not measured more than three times within 48 h of hospitalization were excluded. In addition, among the remaining patients, three patients whose HbA1c levels were not recorded within 2 months of hospitalization were excluded. Eleven patients hospitalized for different periods were counted as individual cases. Finally, medical records of 282 patients were retrospectively analysed. The study protocol was developed in accordance with the tenets of the Declaration of Helsinki and reviewed and approved by the Institutional Review Board of Yeungnam University Hospital (IRB no. 2022-02-011).

### Variables

Patient characteristics, laboratory values, radiologic findings, pneumonia pathogens from sputum culture and treatment strategies were obtained from electronic medical records. Patient characteristics included age, sex, ICU stay and number of days from death. Disease severity was assessed using the Acute Physiologic Assessment and Chronic Health Evaluation (APACHE) II score [14]. Anthropometric and laboratory results included body mass index (BMI), white blood cell (WBC) count, haemoglobin (Hb) level, platelet (Plt) count, C-reactive protein (CRP) level, blood urea nitrogen (BUN) level, creatinine level, estimated glomerular filtration rate (eGFR) and lactate level. Radiological findings included unilateral pneumonia, bilateral pneumonia and multiple ground-glass opacities. Treatment strategies included invasive mechanical ventilation (IMV), continuous renal replacement therapy (CRRT), extracorporeal membrane oxygen (ECMO), antibiotics and glucocorticoids.

### Assessment of glucose profiles

The presence of diabetes, degree of usual blood glucose control based on HbA1c level, and acute hyperglycaemia based on GV were assessed. Diabetes was diagnosed if HbA1c level was ≥6.5% or if it was previously diagnosed by an endocrinologist [15]. The high and low HbA1c levels were classified using a cut-off value of 7.5% [16,17].

We obtained all BST values that were measured within 48 h of admission. GV was measured using the coefficient of variation (CV) calculated from all available BST values [18]. CV refers to the value dividing the standard deviation (SD) of all BST values by the average of those values. Referring to previous studies, high GV was defined as a CV ≥ 36% and low GV as a CV < 36% [19,20]. The equations for the CV and SD are as follows:

$$\mathrm{SD}\;=\sqrt{\frac{\sum {\left(X_{i}-x\right)}^{2}}{k-1}},\;\;\mathrm{CV}=\;\frac{s}{x}$$

(*X_i_* = individual observation, *x* = mean of observations, *k* = number of observations) (*s* = standard deviation, *x* = mean of observations).

The mean, minimal and maximal glucose levels were calculated based on all BST values measured within 48 h of hospitalization, regardless of the number of times.

### Outcomes

The primary outcome was the 28-day mortality post-hospitalization. The secondary outcome was prolonged ICU stay (>14 days) [21].

### Statistical analysis

All analyses were performed using SPSS version 28 (IBM Corp. Armonk, NY). An independent *t*-test was used to compare the groups with continuous variables. The chi-square test was used to compare categorical variables. Kaplan–Meier analysis was performed to compare mortality rates between high GV and low GV subgroups according to the presence of diabetes and HbA1c levels ≥ 7.5% and < 7.5%. The factors affecting 28-days mortality and prolonged ICU stay were investigated using Cox proportional regression analysis and logistic regression analysis, respectively. For the Cox proportional regression analysis, the time variable was 28 days or until death, whichever came first. Statistical significance was set at a *p* value < .05.

## Results

### Baseline characteristics of the patients

The baseline clinical characteristics of the patients (*n* = 282) are summarized in Table 1. The mean age was 60.6 years (male: female ratio, 2.5:1). A total of 160 patients (56.7%) had DM, and they all had type 2 diabetes. The patients were treated in the ICU for an average of 12.4 days. The mean number of BST measurements in 282 patients was 8.26 times. The mean APACHE score of all patients was 25.3. Among all patients, 89 (31.6%) died within 28 days, and their mean survival time was 11.5 days. Compared with living patients, deceased patients were older (67.7 ± 0.9 *vs.* 70.7 ± 1.05, *p* = .045) and had higher APACHE score (23.8 ± 0.5 *vs*. 28.6 ± 0.8, *p* < .001). The deceased patients showed lower Hb level and eGFR and higher CRP, BUN, and lactate levels than living patients (all *p* < .05). The presence of diabetes, HbA1c level, and mean-, minimal-, and maximal-glucose levels did not differ between living and deceased patients; however, the CV (39.6 ± 1.8 *vs*. 46.5 ± 2.6, *p* = .031) and proportion of high GV (46.6 *vs.* 60.7%, *p* = .028) was significantly higher in patients who died in 28 days.

**Table 1.** Baseline characteristics of patients with pneumonia among living and deceased patients.

	Living (*n* = 193)	Deceased (*n* = 89)	*p* Value
Patient characteristics			
Age (years)	67.7 ± 0.9	70.7 ± 1.1	.045
Sex, Male (*n*, %)	140 (72.5%)	62 (69.7%)	.619
ICU stay (days)	13.7 ± 1.8	9.6 ± 0.7	.139
Death days (days)	NA	11.5 ± 0.8	NA
APACHE	23.8 ± 0.5	28.6 ± 0.8	<.001
Anthropometrics and Laboratory results			
BMI (kg/m^2^)	23.0 ± 0.3	22.3 ± 0.5	.127
WBC (10^3^ cells/cc^3^)	13.0 ± 0.6	13.3 ± 0.8	.759
Hb (10^3^ cells/cc^3^)	10.9 ± 0.2	10.2 ± 0.2	.006
Plt (10^9^ cells/L)	224.9 ± 8.0	211.1 ± 14.6	.373
CRP (mg/dL)	14.6 ± 0.8	18.0 ± 1.1	.013
BUN (mg/dL)	26.1 ± 1.3	38.2 ± 3.1	<.001
CRE (mg/dL)	1.7 ± 0.2	1.9 ± 1.9	.484
eGFR	74.2 ± 3.8	55.4 ± 4.6	.004
Lactate (mmol/L)	2.2 ± 0.1	4.1 ± 0.4	<.001
Glucose profile			
Diabetes mellitus (*n*, %)	109 (56.5%)	51 (57.3%)	.896
HbA1c (%)	6.7 ± 0.1	6.6 ± 0.1	.794
High HbA1c (≥7.5%)	41 (21.2%)	17 (19.1%)	.679
Low HbA1c (<7.5%)	152 (78.7%)	72 (80.9%)	
CV	39.6 ± 1.8	46.5 ± 2.6	.031
High GV (CV ≥ 36%)	90 (46.6%)	54 (60.7%)	.028
Low GV (CV < 36%)	103 (53.4%)	35 (39.3%)	
Mean glucose level (mg/dL)	183.3 ± 4.1	192.6 ± 6.3	.207
Minimal glucose level (mg/dL)	127.3 ± 2.8	124.9 ± 4.9	.650
Maximal glucose level (mg/dL)	249.4 ± 6.6	271.4 ± 9.9	.064

Data are presented as mean ± *SD* and *N* (%).

ICU: intensive care unit; APACHE: Acute Physiology and Chronic Health Evaluation; BMI: body mass index; WBC: white blood cell; Hb: haemoglobin; Plt: platelet; CRP: C-reactive protein; BUN: blood urea nitrogen; CRE: creatinine; eGFR: estimated glomerular filtration rate; HbA1c: haemoglobin A1c; CV: coefficient of variation.

The radiological findings, pathogens, and treatment for pneumonia are summarized in Table 2. Bilateral pneumonia accounted for approximately 98% of both living and deceased patients. The pathogen-causing pneumonia was identified in approximately 60% of patients, and the frequency of isolated pathogens did not differ between living and deceased patients. *Acinetobacter baumannii* was most frequent, followed by *Staphylococcus aureus*, *Klebsiella pneumoniae*, and *Pseudomonas aeruginosa.* These pathogens are the major isolates of hospital-acquired pneumonia in Asia [22]. The treatment strategies (IMV, CRRT, ECMO, antibiotics, and glucocorticoids) did not differ between the living and deceased patients.

**Table 2.** Radiologic findings, pathogen and treatment of pneumonia among living and deceased patients.

	Living (*n* = 193)	Deceased (*n* = 89)	*p* Value
Radiologic findings			
Unilateral pneumonia	3 (1.6%)	1 (1.1%)	1.000
Bilateral pneumonia	190 (98.4%)	88 (98.9%)	1.000
Multiple ground-glass opacities	83 (43.0%)	35 (39.3%)	.560
Pathogen			
Pathogen unknown	81 (42.0%)	33 (37.1%)	.514
Pathogen identified	112 (58.0%)	56 (62.9%)	–
Gram-positive			
*Staphylococcus aureus*	44 (22.8%)	28 (31.5%)	.121
Gram-negative			
*Acinetobacter baumannii*	59 (30.6%)	30 (33.7%)	.598
*Klebsiella pneumonia*	22 (11.4%)	12 (13.5%)	.617
*Pseudomonas aeruginosa*	23 (11.9%)	5 (5.6%)	.100
*Stenotrophomonas maltophilia*	10 (5.2%)	4 (4.5%)	.730
Others	10 (5.2%)	8 (9.0%)	.224
Treatment			
IMV	149 (77.2%)	70 (78.7%)	.786
CRRT	16 (17.2%)	12 (13.5%)	.175
ECMO	2 (1.0%)	0 (0%)	1.000
Antibiotics	190 (98.4%)	88 (98.9%)	1.000
Glucocorticoid	73 (37.8%)	42 (47.2%)	0.137

IMV: invasive mechanical ventilation; CRRT: continuous renal replacement therapy; ECMO: extracorporeal membrane oxygenation.

### Association of diabetes, HbA1c level, and GV with 28-day mortality

**Figure 1.**
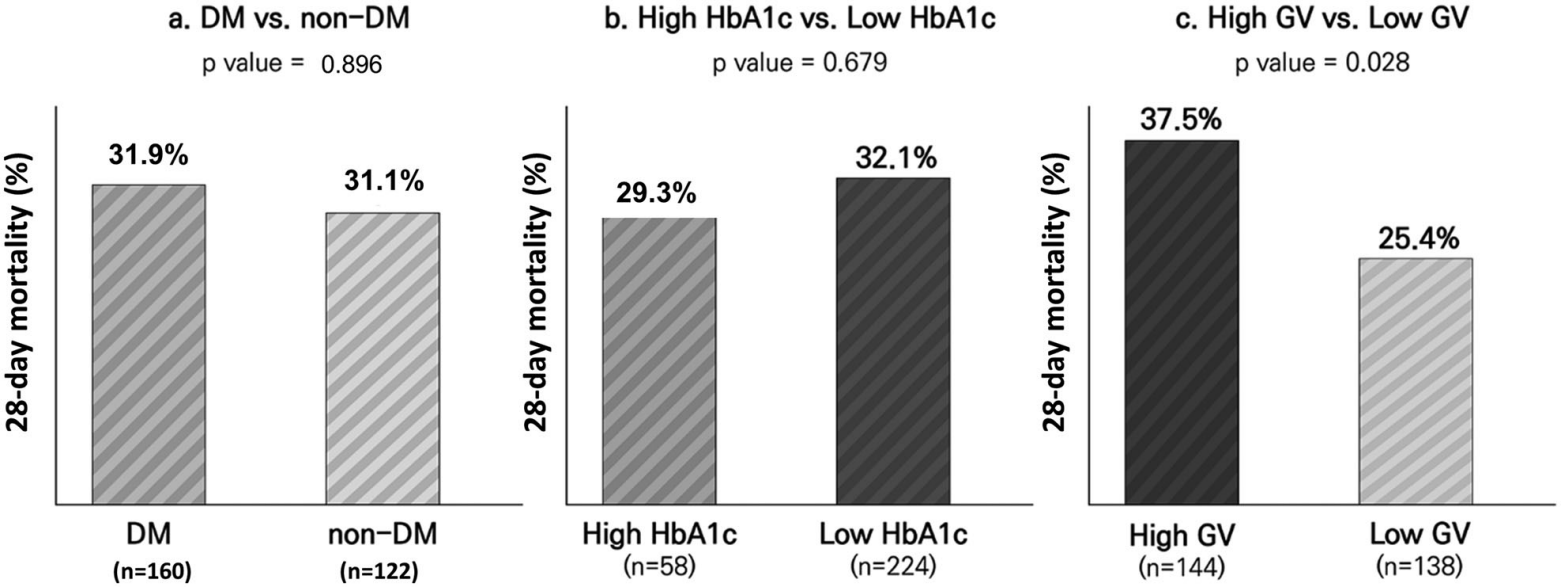
Comparison of 28-day mortality between (a) patients with and without diabetes, (b) patients with high HbA1c levels (≥7.5%) and low HbA1c levels (<7.5%), (c) patients with high GV (CV ≥ 36%) and low GV (CV < 36%). GV: glycaemic variability; HbA1c: haemoglobin A1c.

The association between diabetes, HbA1c, and GV and 28-day mortality is presented in Figure 1. The 28-day mortality rates were not different between patients with and without DM (31.9 and 31.1%, respectively; *p* = .896) and patients with high and low HbA1c levels (29.3 and 32.1%, respectively; *p* = .679). However, the 28-day mortality rate was significantly higher in patients with high GV than in those with low GV (37.5 and 25.4%, respectively; *p* = .028).

**Figure 2.**
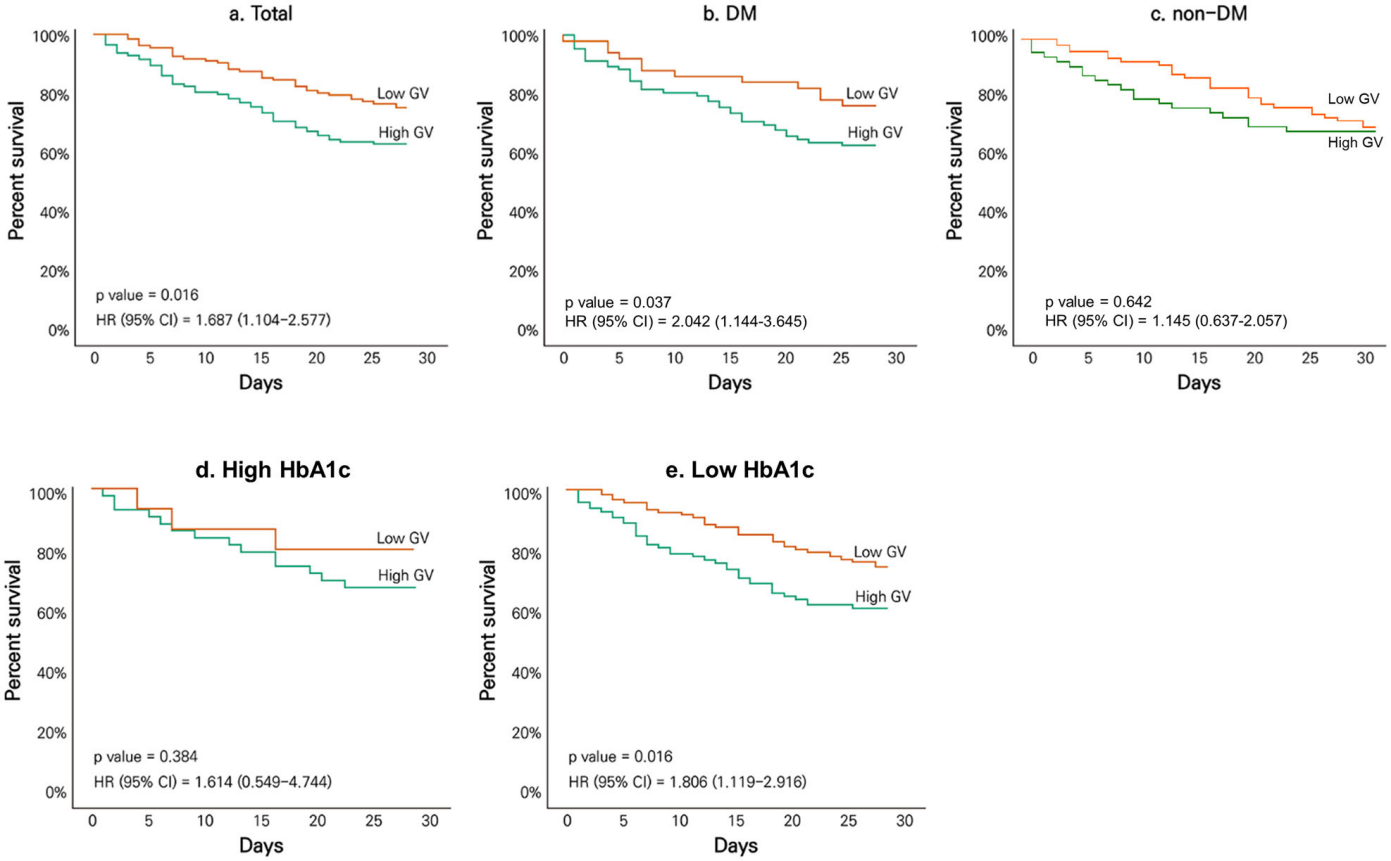
Kaplan–Meier curve for 28-day survival according to GV among (a) total patients, (b) patients with DM, (c) patients without DM, (d) patients with high HbA1c level (≥7.5%), and (e) patients with low HbA1c level (<7.5%). DM: diabetes mellitus; HbA1c: haemoglobin A1c; GV: glycaemic variability; CV: coefficient of variation; HR: hazard ratio; CI: confidence interval.

The effects of GV on 28-day mortality among all patients and subgroups of DM, non-DM, high HbA1c and low HbA1c are shown in Figure 2. Among all patients, the mortality risk was significantly higher in those with high GV than in those with low GV (hazard ratio [HR], 1.687; 95% confidence interval [CI], 1.104–2.577, *p* = .016). In the DM subgroup, high GV showed an increased mortality risk with statistical significance (HR, 2.042; 95% CI, 1.144–3.645, *p* = .037), but not in the non-DM subgroup (*p* = .642). In the high HbA1c subgroup, there was no significant difference in mortality risk between the high and low GV (*p* = .384). Conversely, in the low HbA1c subgroup, the mortality risk was significantly higher in those with high GV than in those with low GV (HR, 1.806; 95% CI, 1.119–2.916; *p* = .016).

### Association of diabetes, HbA1c level, and GV with prolonged ICU stay

**Figure 3.**
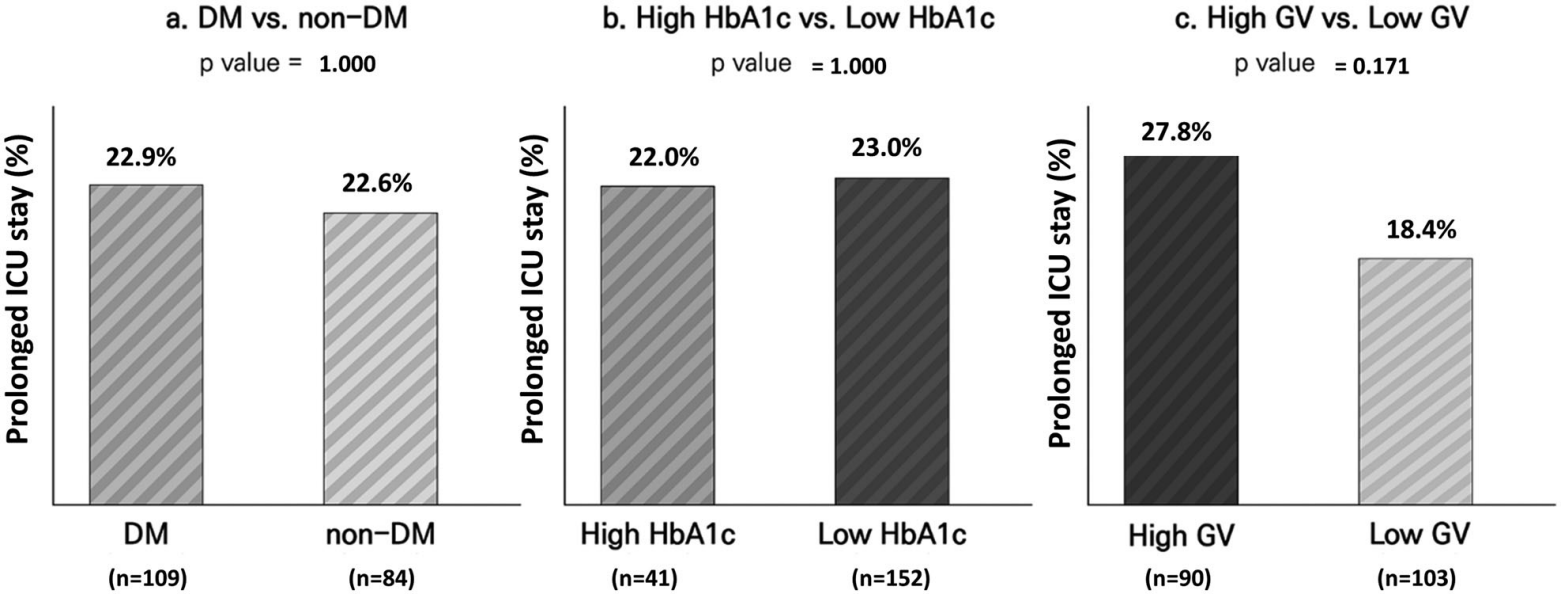
Comparison of prolonged ICU stay (>14 days) between (a) patients with and without diabetes, (b) patients with high HbA1c levels (≥7.5%) and low HbA1c levels (<7.5%), and (c) patients with high GV (CV ≥36%) and low GV (CV <36%). GV: glycaemic variability; HbA1c: haemoglobin A1c; ICU: intensive care unit.

The association between diabetes, HbA1c, and GV with prolonged ICU stay among living patients is shown in Figure 3. The prevalence of prolonged ICU stay was not different between patients with and without diabetes (22.9 and 22.6%, respectively; *p* = 1.000) or patients with high and low HbA1c levels (22.0 and 23.0%, respectively; *p* = 1.000). Although ICU stay was frequently prolonged in patients with high GV compared to those with low GV, this difference was not statistically significant (22.8 and 18.4%, respectively; *p* = .171).

### Risk factors for 28-day mortality and prolonged ICU stay

Cox proportional regression and logistic regression analyses were performed to analyse the factors affecting 28-day mortality and ICU stay (Table 3). Age, sex, APACHE score, CRP level, eGFR, HbA1c level, CV, mean glucose level and treatment strategies (IMV, CRRT, ECMO, antibiotics and glucocorticoids) were considered as covariates. After adjustment, the APACHE score (HR, 1.09; 95% CI, 1.05–1.13; *p* < .001), CRP (HR, 1.02; 95% CI, 1.00–1.04; *p* = .031), and CV (HR, 1.01; 95% CI, 1–1.02; *p* = .04) were significant risk factors, and IMV (HR, 0.56; 95% CI, 0.31–0.99; *p* = .046) was a significant protective factor for 28-day mortality. With respect to prolonged ICU stay, after adjustment, CV (HR, 1.02; 95% CI, 1.00–1.04; *p* = .04) was the only risk factor.

**Table 3.** Risk factors for 28-day mortality and prolonged ICU stay (>14 days).

	28-day mortality^a^		Prolonged ICU stay^b^	
	Adj. HR (95% CI)	Adj. *p* value	Adj. OR (95% CI)	Adj. *p* value
Age	1.01 (0.99–1.03)	.278	1 (0.97–1.03)	.808
Sex : female *vs.* male	1.06 (0.66–1.70)	.813	0.92 (0.41–2.08)	.845
APACHE	1.09 (1.05–1.13)	<.001	0.95 (0.9–1)	.071
CRP	1.02 (1.00–1.04)	.031	0.97 (0.93–1)	.089
eGFR	1.00 (0.99–1.00)	.489	1 (0.99–1)	.283
HbA1c	0.93 (0.78–1.12)	.472	0.71 (0.5–1)	.052
CV	1.01 (1.00–1.02)	.04	1.02 (1–1.04)	.04
Mean glucose level	1.00 (0.99–1.00)	.138	1 (0.99–1.01)	.716
IMV	0.56 (0.31, 0.99)	.046	1.41 (0.57–3.48)	.46
CRRT	2.09 (0.99–4.42)	.053	0.24 (0.04–1.52)	.13
ECMO	0 (0, inf)	.994	3.01 (0.1–92.34)	.528
Antibiotics	1.02 (0.14, 7.51)	.985	0.6 (0.05–7.76)	.696
Glucocorticoid	1.52 (0.98-2.34)	.061	1.07 (0.5–2.26)	.865

^a^ Cox regression analysis.

^b^ logistic regression analysis was conducted.

HR: hazard ratio; OR: odds ratio; CI: confidence interval; APACHE: Acute Physiology and Chronic Health Evaluation; CRP: C-reactive protein; eGFR: estimated glomerular filtration rate; HbA1c: haemoglobin A1c; CV: coefficient of variation; IMV: invasive mechanical ventilation; CRRT: continuous renal replacement therapy; ECMO: extracorporeal membrane oxygenation.

## Discussion

In this study, we evaluated the effect of GV (based on a CV of 36%) within the first 48 h on 28-day mortality and prolonged ICU stay among patients admitted to the ICU due to pneumonia. The 28-day mortality rate was significantly higher in patients with a high GV than in those with a low GV (37.5 *vs.* 25.4%, *p* = .028). Particularly, high GV increased the risk of 28-day mortality in patients with DM or low HbA1c levels (<7.5%). After adjusting for the severity of illness and treatment strategy, CV was an independent predictive factor for 28-day mortality and prolonged ICU stay. Thus, we demonstrated that early phase GV should be managed in critically ill patients.

Previous studies have shown that intermittent hyperglycaemia has more harmful effects than chronic hyperglycaemia in patients with type 2 DM [23]. These effects are related to oxidative stress and endothelial dysfunction [24,25]. Increased GV activates protein kinase C and adhesion molecules, and oxidative stress caused by this process induces apoptosis of endothelial cells [26,27]. Moreover, increased reactive oxygen species in the mitochondrial electron transport chain cause DNA damage, increase polyadenosyl ribose phosphate, form nuclear factor-κB and reduce glyceraldehyde 3-phosphate dehydrogenase [28]. As a result, multiple pro-inflammatory pathways are activated, inducing ischaemia, which leads to angiogenesis [29].

There was no difference in the 28-day mortality and prolonged ICU stay between patients with and without DM. This can be explained by the adaptation of patients with diabetes to chronic hyperglycaemia, resulting in an increased threshold for stress hyperglycaemia-related adverse effects [12,30,31]. However, the risk of DM on the severity and prognosis of respiratory diseases has been emphasized and therefore should not be underestimated [32,33]. In addition, newly discovered hyperglycaemia is an important marker of in-hospital mortality rate and longer length of stay than HbA1c level [30,34]. Taken together, the presence of diabetes or HbA1c alone is insufficient to predict outcomes in critically ill patients. Therefore, the management of GV should be considered to achieve a better prognosis.

In patients with high GV, the 28-day mortality risk was 1.7 times higher than that in those with low GV, indicating that high GV was an independent risk factor for 28-day mortality. This is consistent with a recent study showing that high early GV independently increased 30-day mortality by 1.6 times in patients with sepsis [12]. In addition, among patients with diabetes, high GV doubled the risk of 28-day mortality. The impact of GV among patients without diabetes was invalid in our study, but in another study, high GV significantly increased mortality among patients without diabetes or those in the euglycemic range [35,36]. In the low HbA1c subgroup, the mortality risk in those with high GV was 1.8 times higher than that in those with low GV. In this subgroup, both patients without diabetes and those with well-controlled diabetes were included. The reason for this difference is unclear, but it is presumed that patients with high HbA1c levels are resistant to cellular and microvascular complications, as they are exposed to long-term uncontrolled hyperglycaemia [30,37,38]. These findings imply that high GV is associated with death in patients admitted to the ICU due to pneumonia, and patients with high GV, especially those with low HbA1c levels irrespective of diabetes, might be more carefully treated.

This study had some limitations. First, we retrospectively analysed patients admitted to the ICU at a single centre. Therefore, this may be a limitation when generalizing the results of this study to other nationalities or races. Second, since GV was measured using all available BST results instead of continuous glucose monitoring (CGM), there is a limitation compared to the accuracy of GV using CGM [39,40]; moreover, BST measurements were not conducted the same number of times. Therefore, it should be noted that somewhat inaccurate GV may have been used in the study. Third, conducting research with more patients is expected to lead to new and meaningful results. Fourth, this study did not investigate the usual drug use history of patients, such as insulin, oral hypoglycaemic agents and statins. Lastly, various treatments, including nutritional supply and insulin may have affected GV [41]. Despite these limitations, this study had some strengths. This study not only showed the effect of GV on 28-day mortality and prolonged ICU stay in critically ill patients with pneumonia, but also evaluated its effect among diabetes, non-diabetes, high HbA1c level and low HbA1c level subgroups. We suggest that a high GV can be deleterious, especially in patients with diabetes or low HbA1c levels. As pneumonia is one of the most common single diseases leading to hospitalization of patients in the ICU [42], these findings could be usefully applied in clinical practice. The association of GV and lung function is still unclear; therefore, further studies are needed to identify the effect of GV on various respiratory diseases.

## Conclusions

Early GV is a useful predictor of 28-day mortality and prolonged ICU stay. Strategies to reduce GV may increase favourable outcomes when treating critically ill patients with pneumonia.

## Data Availability

The data that support the findings of this study are available on request from the corresponding author, SMC and JSM. The data are not publicly available due to their containing information that could compromise the privacy of research participants.

## References

[1] Papatheodorou K, Banach M, Edmonds M, et al. Complications of diabetes. J Diabetes Res. 2015;2015:189525. 26247036 10.1155/2015/189525 PMC4515299

[2] Rajasurya V, Gunasekaran K, Surani S. Interstitial lung disease and diabetes. World J Diabetes. 2020;11(8):351–357. 32864047 10.4239/wjd.v11.i8.351 PMC7438183

[3] Ljubić S, Metelko Ž, Car N, et al. Reduction of diffusion capacity for carbon monoxide in diabetic patients. Chest. 1998;114(4):1033–1035. 9792573 10.1378/chest.114.4.1033

[4] Sinha S, Guleria R, Misra A, et al. Pulmonary functions in patients with type 2 diabetes mellitus & correlation with anthropometry & microvascular complications. Indian J Med Res. 2004;119:66–71. 15055485

[5] Ehrlich SF, Quesenberry CP Jr, Van Den Eeden SK, et al. Patients diagnosed with diabetes are at increased risk for asthma, chronic obstructive pulmonary disease, pulmonary fibrosis, and pneumonia but not lung cancer. Diabet Care. 2010;33(1):55–60. 10.2337/dc09-0880 PMC2797986 19808918

[6] Geerlings SE, Hoepelman AI. Immune dysfunction in patients with diabetes mellitus (DM). FEMS Immunol Med Microbiol. 1999;26(3–4):259–265. 10575137 10.1111/j.1574-695X.1999.tb01397.x

[7] Kim EJ, Ha KH, Kim DJ, et al. Diabetes and the risk of infection: a national cohort study. Diabetes Metab J. 2019;43(6):804–814. 31701687 10.4093/dmj.2019.0071 PMC6943267

[8] Falguera M, Pifarre R, Martin A, et al. Etiology and outcome of community-acquired pneumonia in patients with diabetes mellitus. Chest. 2005;128(5):3233–3239. 16304267 10.1378/chest.128.5.3233

[9] Krinsley JS. Glycemic variability: a strong independent predictor of mortality in critically ill patients. Critic Care Med. 2008;36(11):3008–3013. 10.1097/CCM.0b013e31818b38d2 18824908

[10] Silveira L, Basile-Filho A, Nicolini E, et al. Glycaemic variability in patients with severe sepsis or septic shock admitted to an Intensive Care Unit. Intensive Crit Care Nurs. 2017;41:98–103. 28318952 10.1016/j.iccn.2017.01.004

[11] Egi M, Bellomo R, Stachowski E, et al. Variability of blood glucose concentration and short-term mortality in critically ill patients. J Am Soc Anesthesiol. 2006;105(2):244–252. 10.1097/00000542-200608000-00006 16871057

[12] Chao WC, Tseng CH, Wu CL, et al. Higher glycemic variability within the first day of ICU admission is associated with increased 30-day mortality in ICU patients with sepsis. Ann Intensive Care. 2020;10(1):1–10. 32034567 10.1186/s13613-020-0635-3 PMC7007493

[13] Bagshaw SM, Bellomo R, Jacka MJ, et al. The impact of early hypoglycemia and blood glucose variability on outcome in critical illness. Crit Care. 2009;13(3):R91–10. 19534781 10.1186/cc7921 PMC2717463

[14] Knaus WA, Draper EA, Wagner DP, et al. II: a severity of disease classification system. Crit Care Med. 1985;13(10):818–829. 3928249

[15] Kim MK, Ko SH, Kim BY, et al. 2019 Clinical practice guidelines for type 2 diabetes mellitus in Korea. Diabetes Metab J. 2019;43(4):398–406. 31441247 10.4093/dmj.2019.0137 PMC6712226

[16] Ali A, Iqbal F, Taj A, et al. Prevalence of microvascular complications in newly diagnosed patients with type 2 diabetes. Pak J Med Sci. 2013;29(4):899. 24353655 10.12669/pjms.294.3704 PMC3817776

[17] Liu Z, Fu C, Wang W, et al. Prevalence of chronic complications of type 2 diabetes mellitus in outpatients-a cross-sectional hospital based survey in urban China. Health Qual Life Outcomes. 2010;8(1):1–9. 20579389 10.1186/1477-7525-8-62 PMC2906445

[18] Umpierrez GE, Kovatchev BP. Glycemic variability: how to measure and its clinical implication for type 2 diabetes. Am J Med Sci. 2018;356(6):518–527. 30447705 10.1016/j.amjms.2018.09.010 PMC6709582

[19] Monnier L, Colette C, Wojtusciszyn A, et al. Toward defining the threshold between low and high glucose variability in diabetes. Diabetes Care. 2017;40(7):832–838. 28039172 10.2337/dc16-1769

[20] Ceriello A, Monnier L, Owens D. Glycaemic variability in diabetes: clinical and therapeutic implications. Lancet Diabetes Endocrinol. 2019;7(3):221–230. 30115599 10.1016/S2213-8587(18)30136-0

[21] Arabi Y, Venkatesh S, Haddad S, et al. A prospective study of prolonged stay in the intensive care unit: predictors and impact on resource utilization. Int J Qual Health Care. 2002;14(5):403–410. 12389806 10.1093/intqhc/14.5.403

[22] Chung DR, Song JH, Kim SH, et al. High prevalence of multidrug-resistant nonfermenters in hospital-acquired pneumonia in Asia. Am J Respir Crit Care Med. 2011;184(12):1409–1417. 21920919 10.1164/rccm.201102-0349OC

[23] Ceriello A, Esposito K, Piconi L, et al. Oscillating glucose is more deleterious to endothelial function and oxidative stress than mean glucose in normal and type 2 diabetic patients. Diabetes. 2008;57(5):1349–1354. 18299315 10.2337/db08-0063

[24] Monnier L, Mas E, Ginet C, et al. Activation of oxidative stress by acute glucose fluctuations compared with sustained chronic hyperglycemia in patients with type 2 diabetes. JAMA. 2006;295(14):1681–1687. 16609090 10.1001/jama.295.14.1681

[25] Buscemi S, Re A, Batsis J, et al. Glycaemic variability using continuous glucose monitoring and endothelial function in the metabolic syndrome and in type 2 diabetes. Diabetic Medicine. 2010;27(8):872–878. 20653743 10.1111/j.1464-5491.2010.03059.x

[26] Taya N, Katakami N, Mita T, et al. Associations of continuous glucose monitoring-assessed glucose variability with intima-media thickness and ultrasonic tissue characteristics of the carotid arteries: a cross-sectional analysis in patients with type 2 diabetes. Cardiovasc Diabetol. 2021;20(1):1–15. 33947398 10.1186/s12933-021-01288-5 PMC8097791

[27] Quagliaro L, Piconi L, Assaloni R, et al. Intermittent high glucose enhances ICAM-1, VCAM-1 and E-selectin expression in human umbilical vein endothelial cells in culture: the distinct role of protein kinase C and mitochondrial superoxide production. Atherosclerosis. 2005;183(2):259–267. 16285992 10.1016/j.atherosclerosis.2005.03.015

[28] Krishna SVS, Kota SK, Modi KD. Glycemic variability: clinical implications. Indian J Endocrinol Metab. 2013;17(4):611–619. 23961476 10.4103/2230-8210.113751 PMC3743360

[29] Ravi R, Balasubramaniam V, Kuppusamy G, et al. Current concepts and clinical importance of glycemic variability. Diabetes Metab Syndr. 2021;15(2):627–636. 33743360 10.1016/j.dsx.2021.03.004

[30] Lu J, Wang C, Cai J, et al. Association of HbA1c with all-cause mortality across varying degrees of glycemic variability in type 2 diabetes. J Clin Endocrinol Metab. 2021;106(11):3160–3167. 34279663 10.1210/clinem/dgab532 PMC8530707

[31] Krinsley JS, Egi M, Kiss A, et al. Diabetic status and the relation of the three domains of glycemic control tomortality in critically ill patients: an international multicenter cohort study. Crit Care. 2013;17(2):R37–17. 23452622 10.1186/cc12547 PMC3733432

[32] Kim MK, Jeon JH, Kim SW, et al. The clinical characteristics and outcomes of patients with moderate-to-Severe coronavirus disease 2019 infection and diabetes in Daegu, South Korea. Diabetes Metab J. 2020;44(4):602–613. 32794386 10.4093/dmj.2020.0146 PMC7453989

[33] Chung SM, Lee YY, Ha E, et al. The risk of diabetes on clinical outcomes in patients with coronavirus disease 2019: a retrospective cohort study. Diabetes Metab J. 2020;44(3):405–413. 32602272 10.4093/dmj.2020.0105 PMC7332325

[34] Mendez CE, Mok K-T, Ata A, et al. Increased glycemic variability is independently associated with length of stay and mortality in noncritically ill hospitalized patients. Diabetes Care. 2013;36(12):4091–4097. 24170754 10.2337/dc12-2430 PMC3836112

[35] Krinsley JS. Glycemic variability and mortality in critically ill patients: the impact of diabetes. J Diabetes Sci Technol. 2009;3(6):1292–1301. 20144383 10.1177/193229680900300609 PMC2787029

[36] Todi S, Bhattacharya M. Glycemic variability and outcome in critically ill. Indian J Crit Care Med. 2014;18(5):285–290. 24914256 10.4103/0972-5229.132484 PMC4047689

[37] Chao HY, Liu PH, Lin SC, et al. Association of in-hospital mortality and dysglycemia in septic patients. PLoS One. 2017;12(1):e0170408. 28107491 10.1371/journal.pone.0170408 PMC5249165

[38] Handa T, Nakamura A, Miya A, et al. The association between hypoglycemia and glycemic variability in elderly patients with type 2 diabetes: a prospective observational study. Diabetol Metab Syndr. 2021;13(1):1–8. 33794984 10.1186/s13098-021-00656-1 PMC8017873

[39] Chung SM, Lee YH, Kim CO, et al. Daytime glycemic variability and frailty in older patients with diabetes: a pilot study using continuous glucose monitoring. J Korean Med Sci. 2021;36(27):e190. 34254474 10.3346/jkms.2021.36.e190 PMC8275461

[40] Ha EY, Chung SM, Park IR, et al. Novel glycemic index based on continuous glucose monitoring to predict poor clinical outcomes in critically ill patients: a pilot study. Front Endocrinol (Lausanne). 2022;13:869451. 35600594 10.3389/fendo.2022.869451 PMC9114696

[41] Chen HS, Wu TE, Jap TS, et al. Beneficial effects of insulin on glycemic control and β-cell function in newly diagnosed type 2 diabetes with severe hyperglycemia after short-term intensive insulin therapy. Diabetes Care. 2008;31(10):1927–1932. 18556343 10.2337/dc08-0075 PMC2551629

[42] AlOtair HA, Hussein MA, Elhoseny MA, et al. Severe pneumonia requiring ICU admission: revisited. J Taibah Univ Med Sci. 2015;10(3):293–299.

